# On the Ophthalmia Which Prevails in the Belgian Army : With Some Remarks on the Eye-Diseases on the Rhine, and Purulent Ophthalmia in General

**Published:** 1836-10

**Authors:** 


ART. VII.—*Ueber die Augenkrankheit, welche in der Belgischen Armee herrscht. Nebst einigen Bemerkungen über die Augenkrankheiten am Rheine, und über Augenblennorrhoeen im Allgemeinen.* Von J. C. JÜNGKEN, u. s. w.—*Berlin*, 1834.

*On the Ophthalmia which prevails in the Belgian Army; with some Remarks on the Eye-Diseases on the Rhine, and Purulent Ophthalmia in general.* By J. C. JÜNGKEN, M.D., Professor of Medicine in the University of Berlin, &c.—*Berlin*, 1834. 4to. pp. 51.

2. *Ueber die Augenkrankheit welche in der Kaiserlich-Russischen activen Armee herrscht.* Von ROMAN TSCHETIRKIN, M.D. &c. *Aus dem Russischen, von* DR. MAGAZINER.—*Kalisch*, 1835. 8vo. pp. 73.

*On the Ophthalmia prevalent in the Imperial Russian Army.* By DR. TSCHETIRKIN. *Translated from the Russian by* DR. MAGAZINER.——*Kalisch*, 1835. 8vo. pp. 73.

MEDICAL practitioners are generally aware that the purulent inflammations of the conjunctiva are the most destructive of all the ophthalmiæ. Many are deprived of sight in this country from the ophthalmia neonatorum; chiefly, we grant, from the disease being neglected. We have never seen a good statistical account of the effects of the Egyptian ophthalmia in the British army, during their abode in Egypt and after their return. We think it is Sir Robert Wilson who states that, before they left Egypt, 200 men had each lost one eye, and 160 were totally blind; an estimate probably much below the truth. We know on good authority, (that of Dr. Vetch,) that, in 1805, the 52d regiment amounted to somewhat above 700 men; that, from August 1805 to August 1806, 636 cases of ophthalmia occurred in that regiment, including relapses; and that, of these, fifty lost both eyes, and forty one eye each. Our readers, however, may well be astounded when we announce to them the fact, that, in the course of only a few years, no fewer than 4000 individuals belonging to the Belgian army entirely lost their sight from purulent ophthalmia, and 10,000 lost each one eye; all young soldiers, and all pensioned by the state.

The Russian army, about the same period, suffered in the same manner, but in a less severe degree, as we learn from the pamphlet of Dr. TSCHETIRKIN.

"The Russian garrison at Warsaw was for the first time invaded by the epidemic ophthalmia in the year 1818. The disease made its appearance upon the 1st of July, and from then till the 1st of October 1,106 had fallen victims to it. Since then it frequently occurred among the soldiers quartered in the Ordinatzkian barracks. In 1833, it attacked the second battalion of sappers, the Galitzkian and Kostromian regiments of chasseurs which lay in the Alexandrine barracks, and again the Polish recruits. The disease attained neither a great degree of malignity, nor was it remarkably contagious, and ceased in harvest after excessive rains. From April until the middle of August, 1833, out of 934 who were ill, ten lost their sight. The ophthalmia of 1834, however, was far more energetic, and reached a high grade of intensity in the garrison at Warsaw, particularly among the gens-d'armes and the Brankian regiment of chasseurs, quartered in the centre of the town. In the year 1834, the number of individuals labouring under ophthalmia in the active army was 8,000; of whom, thirty-five are permanently blind, some others have lost one eye. Of these latter, several recovered so far as to be able to distinguish certain objects. It did not entirely subside, but persisted during the winter of 1834-5, and was observed from time to time, particularly in the month of February." (P. 2.)

A proposal was some time ago made to the Royal College of Surgeons of London, that they should exact from their candidates attendance on an Eye Hospital, and on a course of instruction in eye-diseases; but we believe it fell to the ground. We cannot conceive that, where the interests of humanity were so intimately concerned, such a result could have any reference to matters of finance. At any rate, the facts to which we have just adverted sufficiently prove the importance of the ophthalmological branch of medicine, and the necessity of exacting a knowledge of it from all licensed practitioners.

It was under the painful circumstances above stated that the Belgian government summoned a medical commission to meet at Brussels, for the purpose of investigating the causes and cure of the disease which had proved so destructive; and this commission Dr. Jüngken was invited to join. He set out from Berlin in February, 1834, and investigated the disease in the hospitals at Brussels, Ghent, Antwerp, Mechlin, and Louvain. The work now before us was written at Brussels, and distributed to the Belgian medical officers. There are some questions, both theoretical and practical, in which certainly we differ from Dr. Jüngken; but, on the whole, we consider this production as one of very great merit, and well deserving the study of British surgeons. The work of Dr. Tschetirkin was composed under somewhat similar circumstances. He is an inspecting physician in the Russian army, and was deputed by the medical staff at Warsaw to furnish an account of the ophthalmia there prevalent. It is also a work of merit.

Before proceeding to Brussels, Dr. Jüngken visited Mayence, Coblentz, Bonn, Cologne, and other places on the Rhine, where purulent ophthalmia had been prevalent for a series of years. At several of them he found a slight epidemic ophthalmia; more, however, among the inhabitants than among the soldiery. For many years the disease had reigned in the Catholic school for teachers (*Schullehrer-Seminar,*) at Brühl, near Cologne, and at length reached such a height that the seminary was evacuated. It is while relating the circumstances of this school that Dr. Jüngken's acceptation of the term "Egyptian ophthalmia" first attracts our notice. He found, he tells us, granular conjunctiva in some of the pupils of the school, but no proof of an Egyptian ophthalmia. By this he means (as more clearly appears in the sequel,) a contagious ophthalmia propagated from person to person, and incapable of originating except from a specific poison, identical with that which attacked the British and French armies in Egypt.

Along the left bank of the Rhine, Dr. Jüngken met with very many individuals suffering under puro-mucous ophthalmia and its sequelæ. He considers those individuals to have suffered from catarrhal or catarrho-rheumatic ophthalmia, degenerating into blenorrhœal, which he acknowledges to prove often as dangerous as the true Egyptian ophthalmia.

In his introduction, which occupies twelve pages, Dr. J. remarks that, at a certain degree of severity, all the blenorrhœal ophthalmiæ not merely present similar, but actually the same symptoms, and produce the same consequences. If the disease is not speedily checked and removed, the conjunctiva becomes granular, and this after catarrhal ophthalmia, as well as after the Egyptian, the gonorrhœal, or the ophthalmia of new-born children. According to Dr. J., the granular projections on the surface of the diseased conjunctiva are mucous papillæ.

As to the causes of the ophthalmia which he met with among the military along the Rhine, he considers cold to have been the chief; the patients having been exposed to this while doing duty as sentinels. The greater number were taken into the hospitals directly from parts where they were exposed to cold, and in some cases they were so suddenly affected with the disease that it was necessary to relieve them before their time. In Coblentz, certain stations were particularly pointed out which were peculiarly productive of the disease; and the general opinion there was, that two-thirds of the patients in that town owed the disease to exposure of this sort.

Cold also, added to the inconvenient construction of the building, seems to have been the chief cause of the ophthalmia in the school at Brühl. The building was low and surrounded by stagnant water; the class-rooms were on the ground-floor, with no cellars underneath them, and the floors very cold. Besides, they were too small, compared with the number of pupils; so that the atmosphere within was bad. All the pupils remained for several hours continuously at their lessons; the upper parts of their bodies became heated, while their legs up to above the knees were cold. Close to the class-rooms lies a cold cloister, paved with stone and beset with draughts of air, into which the pupils pass immediately from the class-rooms, and which, in bad weather and unfavorable seasons of the year, forms the only place of recreation in leisure hours. The sleeping rooms in the uppermost floor are very mean, and contain too many beds. Immediately on awaking in the morning, the young people step out into a corridor, also beset with draughts of air, and there wash, not only the face but the whole head, and then go fasting into the church. For a year or two the bad effects of such influences were not so apparent: those whose general health had suffered from their residence in the school were most liable to the ophthalmia. Our readers are probably aware that the same ophthalmia which Dr. J. met at Brühl was long prevalent in Christ's Hospital in London.

Regarding the mode of propagation of purulent ophthalmia, Dr. J. observes that many erroneous views have been entertained. The contagious nature of the Egyptian ophthalmia is well known, but is universally ascribed (adds he,) to a specific poison, which many erroneously suppose may remain for years in the person without losing its contagious power. Dr. J. probably refers to the fact, that a patient labouring under granular conjunctiva, a sequela which often lasts for years, is liable to relapses, during which purulent mucus is again discharged from the conjunctiva, and most assuredly will excite a similar ophthalmia, if applied to a healthy eye. Dr. J. is aware that the ophthalmia neonatorum is in a high degree contagious, producing the same disease in adults by the casual application of the discharge from the eye of the infant.

The following we regard as an important observation: Dr. Jüngken has not unfrequently met with cases of female children, who, in consequence of ascarides or of scrofula, laboured under a mucous discharge from the vagina, and who, by conveying accidentally some of this to the eyes, have brought on purulent ophthalmia, which, both in progress and severity, was exactly similar to a gonorrhœal inflammation of the conjunctiva. In one instance of this sort, the disease originating thus in a child, seven individuals of a family were affected one after the other, and their eyes greatly endangered.

Even those mucous ophthalmiæ which originate in catarrhal or catarrho-rheumatic inflammation of the eyes are not less contagious, according to our author, but spread by transmission of the secretion from one person to another, especially in the crowded abodes of the poor.

Dr. J. is of opinion that the contagiousness of purulent ophthalmia is nowise dependent on the nature or cause of the disease, nor on any specific poison, but on the degree of its development and the severity of the symptoms. With these, he says, the contagiousness rises and falls. The more severe the symptoms, and the more rapid the progress of the complaint, the more contagious does it become. Purulent ophthalmiæ of rapid course exercise their contagious power even on the healthiest eye, if this is touched by the secretion; they lose this activity in proportion as the inflammatory symptoms subside. If these are gone, and only a mucous discharge remains, this proves injurious only to eyes in a state predisposed to disease.

With regard to the quality of the secretion, this is so much the more virulent the more nearly it resembles laudable pus in consistence and colour. When it does so, the very smallest quantity will inoculate the healthiest eye. The more the secretion recedes from pus in colour and consistence, and assumes the appearance of a mild mucus, like white of egg, and is diluted with tears, the more it loses the contagious power: it affects no longer the healthy eye it may happen to touch, and exercises its contagious power only on such eyes as are in a very irritable state, perhaps already slightly inflamed, and thereby strongly predisposed to disease. The granular condition of the conjunctiva is symbolic of the malignancy of the affection, indicating that it is still endowed with the faculty of transmitting the infection to sound eyes. This important fact is fully established by the observations of the Russian military surgeons in 1834.

The predisposing causes may be either constitutional or individual. The constitutional predisposition is most frequent during the prevalence of east or north-east winds, during rapid changes of temperature and weather, in seasons of great heat, and under a thundery atmosphere. The individual predispositions are scrofulous or abdominal disorders, and the causes productive of congestion and irritation about the head and eyes.

Dr. Jüngken closes his introductory remarks with a reference to the analogy between the purulent ophthalmiæ and gonorrhœa. They are analogous in their nature, symptoms, course, consequence, and mode of treatment. "As in gonorrhœa," says he, "the appearances of inflammation must be regarded merely as symptoms, which increase and decrease with the rise and fall of the disease, and may even entirely vanish, while the specific complaint, the flow of mucus from the urethra, continues; so it is with the appearances of inflammation in the purulent ophthalmiæ. They indicate the degree of severity of the disease, and may totally disappear, while the granular state of the conjunctiva and increased flow of mucus continue. That, in the treatment of a gonorrhœa, besides the greatest attention to cleanliness, the setting aside of the inflammatory appearances is the chief object; and that, previously to the total subsidence of these, all local applications to the urethra, of whatever sort, and especially all stimulating, irritating, astringent, and similar means, cannot be suffered, and will decidedly prove hurtful, is known to every well-informed practitioner. The same holds with regard to the treatment of purulent ophthalmia, only in a yet higher degree; insomuch as the eye is a much more sensitive organ than the urethra. And yet, alas! how many cases of purulent ophthalmiæ are treated from the beginning with topical means, and often with means of a contrary tendency to what is expected, entirely from the greater number of practitioners having adopted the preconceived and unfortunate opinion that eye-diseases are especially confined to the organ affected, and therefore ought to be treated with peculiar local means; an opinion which robs thousands of their sight." (P. 11.)

In these remarks there is much truth, mixed, we think, with a considerable share of error. That many eyes are lost in the purulent ophthalmiæ from trusting entirely in applications to the conjunctiva, and that many a surgeon has put out the sight of his patient with *liquor plumbi acetatis* when he meant thereby to save it, we have no doubt. We believe, however, that, to trust to antiphlogistic treatment alone, and to overlook local applications, would also be highly dangerous. We are convinced that the most successful treatment of this class of the ophthalmiæ requires a combined use of different and even opposite means, of general with local applications, of soothing with smarting, of means likely to moderate the flow of blood into the inflamed parts with means fitted to excite a new action in the diseased membrane.

That part of his work which refers to the Belgian ophthalmia, Dr. J. arranges under the heads of Nature, History, Causes, Prognosis, Prevention, and Treatment.

With regard to its nature, it cannot be doubted that it is the same disease which prevailed epidemically in 1813, 14, and 15, in the Prussian armies, which is endemic in Egypt, in the whole of the Oriental countries, the south of Italy, Calabria and Sicily, and in the south of Spain, and which attacked the French and English armies during Napoleon's campaign in Egypt.

Dr. Jüngken considers the granular state of the conjunctiva as the chief and essential symptom. We certainly agree so far with him in believing that, till this symptom be overcome, the disease must be regarded as still existing; and we must say, that the discharging of soldiers with granular conjunctiva, consequent to a first attack of the ophthalmia, is scarcely less indicative of ignorance than of cruelty. An opposite error, however, appears to have prevailed in the Belgian army. "The chief cause," says Dr. J., "through which the disease continues to prevail in this army, and through which it ever and anon reappears, is that there remains in the ranks a considerable number of soldiers in whom the disease is not completely extinguished, but is only smothered: in whom the inflammatory phænomena indeed have disappeared, but the state of granular conjunctiva continues." He farther states that, among the troops which he examined, he did not find a company in which there were not several individuals (sometimes six, eight, or more,) affected with granular conjunctiva; and that, in the whole army, there might be several thousands so circumstanced. If such individuals are exposed to the slightest over-exertion, or indulge in the slightest intemperance, the ophthalmia is immediately renewed: under repeated relapses thus brought on, vision is lost, and the disease goes on to attack new subjects.

We perfectly coincide in Dr. J.'s views of the origin of the ophthalmia in the Belgian army: that it was no product of a poison brought from Egypt, but that it arose solely from recent and local causes,—such as exposure to cold during the night, especially after extreme heat during the day; forced marshes; excessive fatigue; sentinel duty; tight uniforms, and especially tight collars, &c. Dr. J. seems to consider an easy, loose uniform, without any tight binding upon the neck, as peculiarly adapted to prevent soldiers being attacked by the ophthalmia, and with reason asks, what we should say were it proposed to oblige sailors, husbandmen, and others, to perform their several kinds of labour in the tight uniform in which a soldier is forced to perform his?

Dr. Tschetirkin's experience as to the origin of the disease in the Russian army coincides very closely with that of Dr. Jüngken. He tells us that the epidemic followed in the wake of the influenza of 1833. It was of catarrhal origin, and seemed to denote a peculiar morbific atmospheric condition. Hence persons much exposed to atmospheric vicissitudes were most liable to be attacked. Many soldiers who escaped ophthalmia suffered from diarrhœa. Among the local circumstances that favoured its development in Warsaw, the elevated situation of the place, the sandy soil, lofty houses, with foul and narrow streets in which the air was never properly renewed, are to be reckoned. Soldiers were especially predisposed, from barrack malaria, exposure cf the eyes to dust, to an air loaded with moisture from the wet clothes hung up to dry, and to the glare of burnished firearms on parade.

The minute enumeration of the predisposing causes by Dr. Jüngken we would earnestly recommend to the perusal of those intrusted with the direction of medico-military affairs.

We cannot agree with Dr. Jüngken in his notions regarding the secretion from the conjunctiva. He thinks it becomes "corrosive, so that, at the greatest height of the disease, it operates very destructively, exactly like quicklime." He supposes it is the secretion which forms the large ulcers on the cornea, and thus destroys the eye. Now, the ulcers are, to our view, merely the effect of the severe inflammation, and we have no evidence of the discharge possessing any corrosive property. However this may be, to remove the discharge thoroughly and frequently from the eye is a point of practice regarding which there cannot be two opinions. Dr. J. recommends this to be done with lukewarm water, decoction of mallows, or infusion of henbane or belladonna, applied by means of the sponge or the syringe.

Dr. Jüngken very properly scouts the idea of any specific remedy for purulent ophthalmia. His practice, on the whole, we consider as judicious, embracing as it does the following particulars: 1. Plentiful venesection; leeches behind the ears; arteriotomy after venesection, or as a substitute for leeches. 2. Purging, especially with calomel. 3. Friction of the brow and temple with mercurial salve and opium. 4. The internal administration of *aqua lauro-cerasi* and of nitre. 5. Excision of a fold of chemosed conjunctiva. 6. When the pain and swelling have subsided, a solution of one grain of corrosive sublimate in ten or twelve ounces of water, or of two grains of *lapis divinus** in six or eight ounces of water, to be dropt into the eyes every six hours or oftener. 7. When all inflammation and unnatural sensibility of the eye are removed, solutions of zinc, nitrate of silver, and the like.

The last two sets of remedies Dr. J. is too late in commencing. Much advantage is derived from the solution of nitrate of silver in the form of drop, and from that of corrosive sublimate as a lotion, even from the very commencement of the disease. Dr. J. appears also to neglect the use of counter-irritation till the symptoms assume a chronic form.

The treatment laid down by Dr. Tschetirkin is also, on the whole, vigorous and judicious. After free depletion, calomel, in doses of one or two grains every two or three hours, was found beneficial: when symptoms of iritis were present, the calomel was exhibited in combination with digitalis or extract of henbane. The ordinary practice was occasionally deviated from, and with a happy result.

From the close analogy subsisting between ophthalmo-blenorrhœa and gonorrhœa, and from the conjunctival corresponding with the urethral mucous membrane in structure as well as in sympathy, Dr. Reinhard bethought himself that the remedies which answered in the one case might also succeed in the other: he therefore administered, in the early stage of the purulent ophthalmia, after abstraction of blood, the *mistura gonorrhoïca* of Delpech, (a combination of capivi,) and that, it is said, with the most beneficial results.

An assistant surgeon, Dr. Belousowitsch, laboured for several months under a masked intermittent fever, which latterly observed monthly paroxysms. From the 1st April, 1835, instead of the ordinary febrile phænomena, he was affected every evening with inflamed eyelids, together with violent pain in the eye. During the seizure the pulse, at first full, became afterwards accelerated; the appetite failed; and the urine yielded a furfuraceous sediment. Assuming the palpebral affection to be of the nature of ague, Dr. B., after due evacuation of the bowels, took inwardly quinine in union with cinchona and opium, and bathed his eyes with a wash containing two grains of sulphate of quina in two ounces of distilled water. The good effects of this treatment were remarkable. The inflammation of the eyelids rapidly subsided, and was entirely gone in four days. He adopted the same line of practice in a number of cases of purulent ophthalmia subsequently with great advantage, where the pain observed periodic remission and exacerbation, and when the urine was turbid.

Granular conjunctiva Dr. J. treats with nitrate of silver, pyroligneous acid, chloride of lime, and even sulphuric acid; and recommends exercise out of doors. He snips off the granular projections with the scissors. The practice of Dr. Tschetirkin was to destroy the granulations by repeated cauterization with lunar caustic or bluestone. When the escharotic could not be applied in substance, its solution was employed. An ophthalmological friend, in whose observations we have great confi-

* *Lapis divinus s. ophthalmicus,* ex vitrioli, nitri et aluminis partibus anaticis, addita camphora, paratur. *Blancard. Lex. Med.*—Ed.

dence, informs us that the projections called granulations of the conjunctiva, are, in most cases, small cysts or vesicles containing a thickish mucus. The treatment he has found most successful is the making of a small crucial incision with the lancet through each of the prominences. These minute scarifications are to be repeated every second or third day; while daily a small bit of strong red precipitate salve is to be put into the eye, rubbing the upper eyelid over the eyeball, so as to diffuse the salve over the inner surface of the eyelid. We have tried this practice, and have found it successful.

Both the works we have noticed are valuable; but that of Dr. Jüngken is very superior to the other, and possesses much more of an original character. The style is clear and perspicuous; unlike most German writings, the pages are not interlarded with references and quotations; the facts are extremely interesting and important, and the reasonings careful and in general correct.

